# A multimodal approach integrating NK cell-associated gene signatures and pathomics to predict colon adenocarcinoma prognosis

**DOI:** 10.1038/s41598-026-49584-y

**Published:** 2026-04-22

**Authors:** Kaiqiang Yang, Qiange Lin, Jia Zhu, Tao Zhu, Guoxiang Fu

**Affiliations:** 1 Department of Pathology, Sir Run Run Shaw Hospital, Zhejiang University School of Medicine, Hangzhou, 310016 China; 2 School of Medical Technology and Information Engineering, Zhejiang Chinese Medical University, Hangzhou, 310053 China

**Keywords:** Colon adenocarcinoma, Single-cell RNA sequencing, Natural killer cells, Deep learning, Pathomics, Multimodal prognostic model, Biomarkers, Cancer, Computational biology and bioinformatics, Oncology

## Abstract

Single-cell RNA sequencing (scRNA-seq) was used to analyze the GSE161277 dataset to identify candidate NK cell–associated genes. A prognostic model based on NK cell-associated gene signatures was constructed via LASSO regression. Deep learning using the Clustering-constrained Attention Multiple Instance Learning model extracted pathomic features from 458 TCGA-COAD whole-slide images. A multimodal prognostic framework was developed by integrating scRNA-seq, transcriptomic, pathomic, and clinical data. scRNA-seq analysis of the GSE161277 dataset revealed diverse immune and stromal cell populations within the microenvironment, highlighting its cellular heterogeneity. Based on these data, NK cell-associated candidate genes were identified for subsequent prognostic modeling. The NK cell-associated gene signature prognostic model showed high accuracy in predicting overall survival in TCGA COAD cohort, with a concordance index (C-index) of 0.835. The pathology-based prognostic model achieved a C-index of 0.871. The multimodal prognostic framework achieved a C-index of 0.889, outperforming single-modality approaches. This study proposes a multimodal prognostic framework integrating NK cell-associated molecular features, pathomic features, and clinical variables.

**Supplementary Information:**

The online version contains supplementary material available at 10.1038/s41598-026-49584-y.

## Introduction

 Colon adenocarcinoma (COAD), the most common malignant gastrointestinal cancer, ranks among the leading causes of cancer-associated deaths worldwide^1^. In 2020, approximately 1.14 million individuals were diagnosed with COAD, leading to 5.7 million deaths^2,3^. Despite progress in screening and treatment, the high heterogeneity and frequent late-stage diagnosis of COAD contribute to poor prognosis, underscoring the need to explore its molecular mechanisms, tumor microenvironment (TME) characteristics, and develop precise prognostic models^4,5^.

The TME plays a critical role in tumor initiation, progression, and metastasis, with tumor-infiltrating immune cells regulating the balance between anti-tumor immunity and immunosuppression^6^. CD8 + T cells and natural killer (NK) cells exhibit anti-tumor activity, while regulatory T cells promote immune escape^7,8^. NK cells, as key components of the innate immune system, can directly kill tumor cells through perforin/granzyme release without prior antigen sensitization and secrete cytokines such as IFN-γ and TNF-α to regulate immune responses^9^. In COAD, reduced NK cell infiltration density, downregulated activation markers, and impaired cytotoxic function are key mechanisms of tumor immune escape^10^. Single-cell RNA sequencing (scRNA-seq) has revealed TME cellular heterogeneity in COAD, including the diversity of T cells, monocytes, and macrophages^11–13^. However, existing studies have mostly focused on CD8 + T cells, macrophages, and other immune cell subsets, and there is a lack of systematic construction of prognostic models based on NK cell-associated gene signatures^14^.

Deep learning (DL)-based techniques, such as Clustering-constrained Attention Multiple Instance Learning (CLAM), have significantly advanced histopathological image analysis by employing attention mechanisms to identify regions of high diagnostic significance, thereby improving the accuracy of cancer detection and classification^15^. These methods enable the detection of subtle morphological patterns critical for precise diagnosis. Despite progress in tumor research through single-cell omics and pathomics, single-modality data often fail to capture the full complexity of tumors^16^. Multimodal analysis, integrating single-cell omics, transcriptomics, pathomics, and clinical data, offers a comprehensive framework for characterizing tumor features, enhancing prognostic accuracy, and guiding personalized treatment strategies^17^. This approach captures the complex interplay of genetic, cellular, and clinical factors driving tumor behavior. However, studies on multimodal prognostic models for COAD, particularly those combining NK cell-associated genes with DL-based histopathological analysis, remain limited^18^.

The purpose of this study is to identify NK cell-associated genes through scRNA-seq analysis, construct an NK-associated prognostic model, and integrate it with deep learning-based pathomics to establish a multimodal prognostic framework. By integrating single-cell omics, transcriptomics, pathomics, and clinical data, a multimodal prognostic model was constructed to provide novel strategies for personalized COAD treatment, advancing the understanding of NK cells and laying the foundation for innovative diagnostic and therapeutic approaches.

## Materials and methods

### Data sources and processing

The single-cell RNA sequencing dataset (GSE161277, accessed on March 23, 2024) was retrieved from the Gene Expression Omnibus (GEO) database, comprising data from 3 normal tissues and 4 adenoma tissues^19^. Gene expression profiles and corresponding clinical data were retrieved from the TCGA dataset (TCGA-COAD, accessed on May 17, 2024), comprising 41 normal colon tissues and 483 COAD tissues, available at https://tcgadata.nci.nih.gov/tcga/. Additionally, gene expression data from the GSE17538 dataset (accessed on July 1, 2024) were acquired from the GEO database^20^. Protein expression in COAD was verified through immunohistochemical staining in the Human Protein Atlas (HPA, http://www.proteinatlas.org/) online database. A total of 458 whole-slide images (WSIs, accessed on April 18, 2025) were obtained from the TCGA-COAD cohort, with 425 WSIs containing survival data selected for feature extraction and subsequent survival model construction. Since the database is publicly accessible for research purposes, ethical approval was not required.

Single-Cell RNA Sequencing Data Analysis.

The R package “Seurat” (v5.1.0) was used for scRNA-seq data analysis^21^. Sample data were imported and merged into a Seurat object, and the proportion of mitochondrial genes was calculated. Cells were filtered to retain those with nFeature_RNA > 50 and percent.mt < 5. To evaluate the robustness of this filtering criterion, we additionally performed a sensitivity analysis using a stricter threshold (nFeature_RNA > 200 and mitochondrial gene percentage < 5%). Data were normalized using LogNormalize and 2,000 highly variable genes were identified using the “vst” method, followed by data scaling. Dimensionality reduction was performed via principal component analysis (PCA) and visualized. Batch effects were corrected using the Harmony algorithm (v1.2.1), with parameters specified as “IntegrateLayers” with “reduction = pca” and “dims = 1:20” in the integration process. Clustering was conducted based on corrected results with a resolution of 0.5, followed by uniform manifold approximation and projection (UMAP) analysis^22^. Cell types were determined through manual annotation referencing known marker genes, and dual validation was performed using the SingleR package (v2.0.0) combined with the HumanPrimaryCellAtlas and BlueprintEncode reference databases to ensure the accuracy of subpopulation annotation.

Construction and Validation of NK Cell-Associated Prognostic Model.

LASSO regression was performed using the R package “glmnet” to screen variables significantly associated with prognosis and determine their coefficients^23^. When conducting LASSO regression, the key parameters of the “cv.glmnet” function were set as “family = cox”, “alpha = 1 (L1 regularization) ”, and the number of cross-validation folds was “nfolds = 10”. The optimal lambda parameter was selected as “lambda.min” to balance model fit and parsimony. A prognostic model was constructed using the equation:

$$\:\mathbf{r}\mathbf{i}\mathbf{s}\mathbf{k}\:\mathbf{s}\mathbf{c}\mathbf{o}\mathbf{r}\mathbf{e}\:=\:\mathbf{v}\mathbf{a}\mathbf{r}\mathbf{i}\mathbf{a}\mathbf{b}\mathbf{l}\mathbf{e}\:1\:\times\:\:\boldsymbol{\upbeta\:}1\:+\:\mathbf{v}\mathbf{a}\mathbf{r}\mathbf{i}\mathbf{a}\mathbf{b}\mathbf{l}\mathbf{e}\:2\:\times\:\:\boldsymbol{\upbeta\:}2\:+\:...\:+\:\mathbf{v}\mathbf{a}\mathbf{r}\mathbf{i}\mathbf{a}\mathbf{b}\mathbf{l}\mathbf{e}\:\mathbf{n}\:\times\:\:\boldsymbol{\upbeta\:}\mathbf{n}$$

where variables represent the values of respective indicators, and β denotes coefficients obtained from LASSO regression analysis. To address the risk of overfitting, the following strategies were adopted: L1 regularization (LASSO regression) was used for feature selection to automatically eliminate redundant variables and reduce model complexity. The R packages “survminer” and “survival” were utilized to produce survival curves, employing the Kaplan-Meier method to visualize survival differences across risk groups^24,25^. The R package “timeROC” was used to create receiver operating characteristic (ROC) curves to assess the predictive accuracy of the risk score for 1-, 3-, and 5-year overall survival (OS)^26^. A multivariate Cox proportional hazards model was constructed using the R package “rms” through stepwise regression to identify key prognostic factors. A nomogram was developed to visualize survival probability predictions at 1, 2, 3, 5, and 10 years. Calibration curves were employed to evaluate the agreement between predicted and actual values, while the concordance index (C-index) was computed to measure the model’s discriminative performance.

### Gene set variation analysis (GSVA)

GSVA was performed to evaluate gene set enrichment in transcriptomic data^27^. Gene lists were obtained from the Gene Set Enrichment Analysis (GSEA) portal. Functional enrichment scores for each sample were calculated using the GSVA package in R under default parameters. Heatmaps of enrichment results were generated using the R package “pheatmap”. Pearson correlation analysis was conducted to determine correlations^28^. Kyoto Encyclopedia of Genes and Genomes (KEGG) pathway analysis and Gene Ontology (GO) enrichment analysis were performed on the risk scores of 16 NK cell-associated prognostic genes to explore their biological functions^29^.

### Immune cell infiltration analysis and immunotherapy response prediction

The R package “CIBERSORT” was applied to analyze RNA-seq data, estimating the proportions of 22 different infiltrating immune cell types^30^. To forecast potential immunotherapeutic responses, the Tumor Immune Dysfunction and Exclusion algorithm was used to assess the immune escape potential across risk groups^31^. We analyzed the distribution patterns of different clinical subtypes across the risk groups and examined the as-sociation between clinical subtypes and the distribution of NK cell risk groups^32^.

Deep Learning Feature Extraction and Selection.

WSIs were segmented into multiple patches. Using the Python library OpenSlide, tissue regions were divided into non-overlapping 256 × 256pixel patches at 20× magnification, and background and necrotic regions were excluded based on tissue region screening thresholds. A modified ResNet50 model, pre-trained on ImageNet, was used to extract feature vectors from these patches^33^. Adaptive average pooling was applied after the third residual block of ResNet50, generating a 1,024-dimensional feature vector for each patch, which was then fused by average pooling.

### CLAM attention heatmap

The CLAM single-branch model (clam_sb) was employed to evaluate and rank patches within WSI tissue regions, assigning attention scores that reflect their contribution to slide-level classification for tumor vs. normal differentiation^34^. The core training and feature extraction parameters are as follows: a truncated ResNet50 encoder (resnet50_trunc) was used for feature extraction, with target patch size of 224 × 224 pixels, batch size of 512, and extracted features stored as HDF5 and PyTorch tensor files. For model training, the initial learning rate was set to 2 × 10⁻⁴ with Adam optimizer and L2 regularization (1 × 10⁻⁵); attention layer dimension was 1024, and dropout rate was 0.25. Maximum training epochs were 200, with early stopping enabled to avoid overfitting. To prevent overfitting, an Early Stopping strategy was implemented with a patience of 20 epochs and a minimum threshold of 50 epochs. A weighted sampling strategy was adopted, with 8 positive/negative patches sampled per batch. The loss function combined cross-entropy loss for bag-level classification and SVM loss for instance-level clustering, with bag loss weight set to 0.7. A weakly supervised validation framework was applied: pseudo-labels were generated by selecting Top-K and Bottom-K patches based on attention scores, constraining feature space separation via instance-level clustering. To visualize model decision-making, unnormalized attention scores of all patches were calculated using the predicted category’s attention branch, converted to percentiles and normalized to the (0,1) range (1 = most predictive, 0 = least informative). Normalized scores were mapped to an RGB color gradient (red = high attention, blue = low attention) and superimposed on patch spatial coordinates to generate attention heatmaps. High-attention regions identified by the model were confirmed by 2 senior pathologists to have clear physiological significance. The model was trained with 10-fold cross-validation on 75% of labeled data, with a fixed random seed (1) for reproducibility, and computations were performed on a CUDA-enabled GPU^35^.

### Random survival forest analysis

Survival data and pathological features were analyzed using R, with the “randomForestSRC” package employed to construct a random survival forest model for risk prediction in COAD patients^36^. Data were standardized and split into 70% training and 30% testing sets, with a random seed set to ensure reproducibility of results. The hyperparameters of the model were set as follows: “ntree = 500” (number of decision trees), “mtry = ncol (X scaled)/3” (number of features randomly selected for each tree), “nodesize = 5” (minimum number of samples for terminal nodes), and “splitrule = logrank” (splitting rule). Model performance was evaluated using the C-index and ROC curves, and Kaplan-Meier survival curves were generated to distinguish high-risk and low-risk groups^37^.

### Construction of multimodal prognostic model

The specific integration process of the multimodal model is as follows: First the NK cell-associated risk prognostic model was obtained using LASSO coefficient weighted scoring; Second the pathology-related risk prediction model was constructed through random forest; Last the NK cell risk score (continuous variable), the predicted probability of the pathology risk model (dichotomous variable), and clinical variables were combined to form a multimodal integrated feature set. The Bootstrap method (1000 resamplings) was used to evaluate the C-index, calibration curves (1/2/3/5/10 years), ROC curves, and area under the curve (AUC) values of the combined model, and compare them with the single-modal models^38^. Based on the independent prognostic factors of the integrated model, a clinical nomogram was constructed to quantify the contribution weights of each feature (NK cell risk score, pathology risk, clinical variables) to the 1/2/3/5/10-year OS. Statistical comparisons between C-index values of different prognostic models were performed using the compareC package in R, which implements a statistical test for comparing correlated concordance indices derived from the same dataset. Because the compareC method requires paired predictions from the same patients, the comparison analyses were conducted on the subset of patients for whom risk scores from all models were available.

## Results

### Identification of colon cell subtypes

**Fig. 1:**
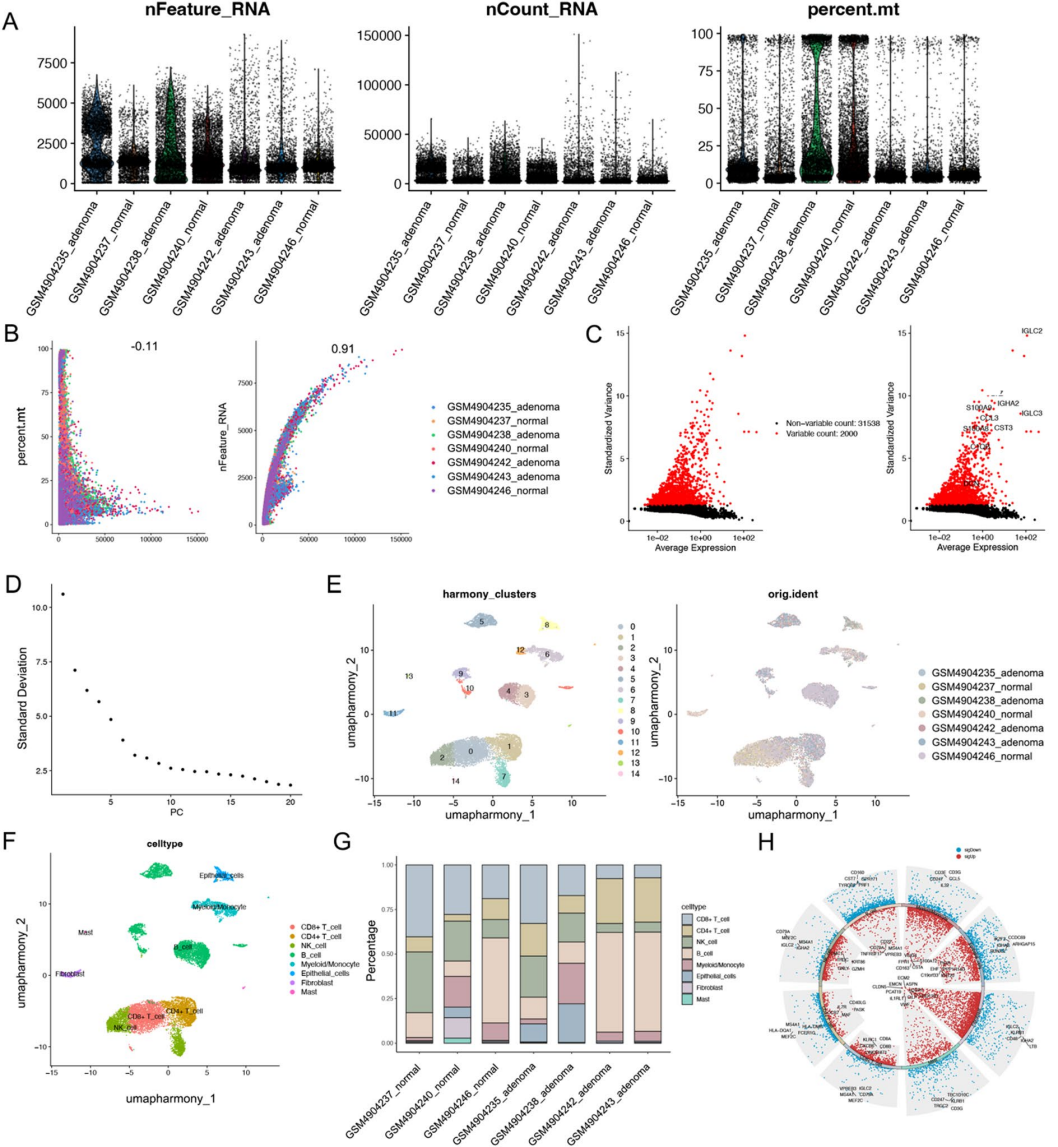
Single-cell RNA (scRNA-seq) sequencing reveals 8 cell clusters with distinct annotations and heterogeneous expression patterns in Colon. (**A**,** B**) A total of 12,042 cells were identified; (**C**) Gene expression variability across all cells. Red dots indicate highly variable genes, while black dots denote genes with stable expression; (**D**,** E**) Dimensionality reduction and clustering identified 15 clusters; (**F**) 8 clusters were annotated as distinct cell types based on their respective marker genes; **(G)** Composition of cell subpopulations in each sample; (**H**) Differential expression of genes across various cell types.

Using single-cell RNA sequencing data from GSE161277, we obtained gene expression profiles from 30,896 cells derived from 4 adenoma samples and 3 normal tissue samples for further analysis (Fig. 1A). Cells were filtered based on nFeature_RNA > 50 and percent.mt < 5, resulting in 12,042 cells for downstream analysis (Fig. 1B). To evaluate whether the permissive quality-control threshold affected downstream results, we additionally performed a sensitivity analysis using stricter filtering criteria (nFeature_RNA > 200 and mitochondrial gene percentage < 5%) (Figure S1). The resulting dataset retained 12,021 cells, with nearly identical QC statistics compared with the original filtering (median genes per cell: 1,196 vs. 1,195; median UMI counts per cell: 3,172 vs. 3,170). These findings indicate that the permissive threshold did not substantially influence the retained cell population or overall cellular composition. Approximately 2,000 highly variable genes were identified, with the top 10 highly variable genes visualized (Fig. 1C). PCA was performed using the top 2,000 variable genes for dimensionality reduction, identifying 15 cell clusters (Fig. 1D and E). By analyzing marker genes, 8 cell clusters were annotated: NK cells, CD4 + T cells, CD8 + T cells, myeloid/monocytes, mast cells, B cells, fibroblasts, and epithelial cells (Fig. 1F). The composition of cell subpopulations varied significantly across samples (Fig. 1G). A volcano plot illustrated the distribution of differentially expressed genes among cell types (Fig. 1H). NK cell clusters were identified based on the expression of canonical NK cell marker genes. Differential expression analysis between NK cell clusters and other immune populations identified a set of NK cell-associated genes for downstream analyses.

### Construction and validation of nk cell-associated prognostic model

**Fig. 2:**
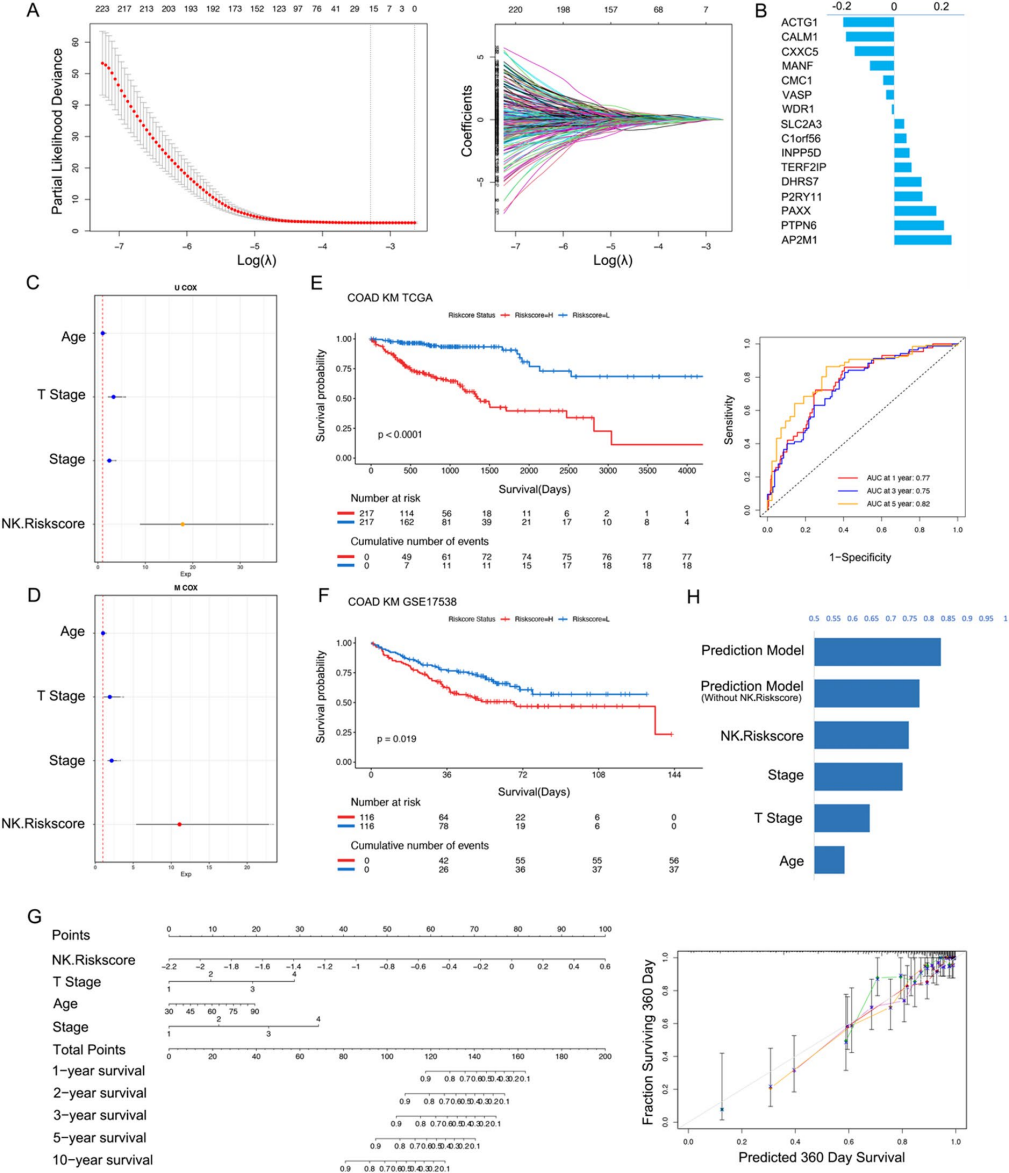
Construction and validation of a natural killer (NK) cell-associated prognostic model using The Cancer Genome Atlas (TCGA) cohort data. (**A**) LASSO regression for overall survival (OS) related genes; (**B**) NK cell-associated prognostic genes and their coefficients; (**C**,** D**) Univariate and multivariate Cox regression analyses for COAD patients; (**E**,** F**) Kaplan-Meier (KM) survival curves and receiver operating characteristic (ROC) curve results for low-risk and high-risk groups; (**G**) Construction of a nomogram and its calibration plots; (**H**) Evaluation of the NK cell-associated prognostic model using the concordance index (C-index).

We extracted 744 feature genes from the NK cell subpopulation for further analysis. To evaluate the risk related to overall survival, a prognostic model was developed by combining LASSO coefficients with RNA expression levels, leading to the identification of 16 NK cell marker genes (Fig. 2A-B). A standardized risk score was calculated for each sample. Univariate and multivariate Cox regression analyses identified the NK cell risk score as an independent prognostic factor (Fig. 2C and D). Patients were stratified into low-risk and high-risk groups based on the median risk score. Kaplan-Meier (KM) analysis revealed that individuals with high-risk scores had significantly shorter OS compared to those with low-risk scores (Fig. 2E). The AUC of ROC curves for OS at 1, 3, and 5 years exceeded 0.751, indicating robust model performance. External validation confirmed that high-risk individuals exhibited significantly shorter OS (Fig. 2F). A nomogram was constructed to visually represent predictive variables, including NK cell risk score, T stage, age, and tumor stage, along with their weights, aiding clinicians in assessing patient prognosis and guiding treatment decisions. The nomogram enabled individualized predictions of COAD prognosis at 1, 2, 3, 5, and 10 years. Calibration curves demonstrated satisfactory agreement between predicted and observed outcomes, indicating high predictive accuracy (Fig. 2G). The C-index of the nomogram model was 0.835 (Fig. 2H).

### Functional enrichment and immune status analysis based on NK risk scores

**Fig. 3:**
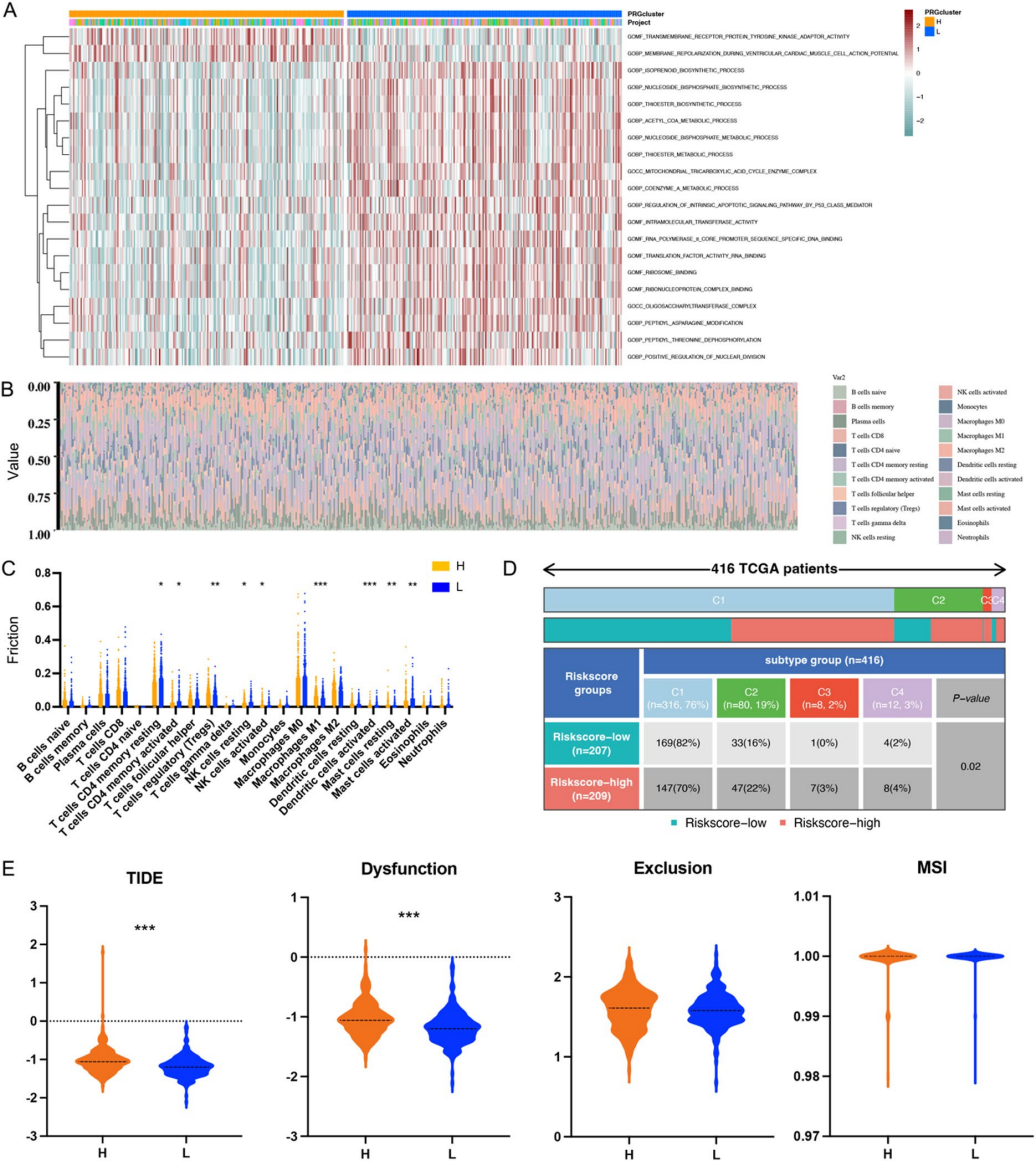
Functional enrichment and immune status analyses. (**A**) Gene Set Variation Analysis (GSVA) functional enrichment map for high-risk and low-risk groups; (**B**) Histogram displaying the percentage of immune cells in each sample; (**C**) Differences in immune cell infiltration between high-risk and low-risk groups. *, *P* < 0.05; **, *P* < 0.01; ***, *P* < 0.001; (**D**) Distribution of clinical subtypes and NK cell risk groups; (**E**) Tumor Immune Dysfunction and Exclusion (TIDE) scores for high-risk and low-risk groups. ***, *P* < 0.001.

Through GSVA, distinct differences were identified across multiple biological processes and signaling pathways when comparing the high-risk group with the low-risk group (Fig. 3A). KEGG pathway and GO enrichment analyses of the 16 NK cell-associated prognostic genes showed significant enrichment in the NF-kappa B signaling pathway, MAPK pathway, and Rap1 pathway, which are core pathways regulating NK cell activation, cytotoxicity, and migration^39–41^(Figure S2). Analysis of TCGA data showed significant variability in immune cell percentages across samples, indicating heterogeneity in immune status (Fig. 3B). Immune cell infiltration analysis identified differences in T cells CD4 memory activated, T cells CD4 memory resting, T cells regulatory, NK cells resting, NK cells activated, Macrophages M1, Dendritic cells activated, Mast cells resting and Mast cells activated between risk groups (Fig. 3C). Visualization of 416 TCGA patients demonstrated associations between clinical subtypes (C1, C2, C3, C4) and NK cell risk group distribution. The subtype proportions were C1 (76%), C2 (19%), C3 (2%), and C4 (3%), with distinct distribution patterns across NK cell risk groups. Statistical analysis confirmed a significant association between clinical subtypes and NK cell risk group distribution (*P* = 0.020), suggesting a potential intrinsic relationship (Fig. 3D). Tumor Immune Dysfunction and Exclusion (TIDE) scores, which evaluate tumor immune response, indicated that the high-risk group had higher TIDE scores, suggesting greater potential for immune escape and reduced benefit from immune checkpoint inhibitors. Immune dysfunction analysis further supported poorer immunotherapy efficacy in the high-risk group. No significant differences were observed in immune exclusion or microsatellite instability between risk groups (Fig. 3E).

### Validation of NK cell-associated prognostic genes

**Fig. 4:**
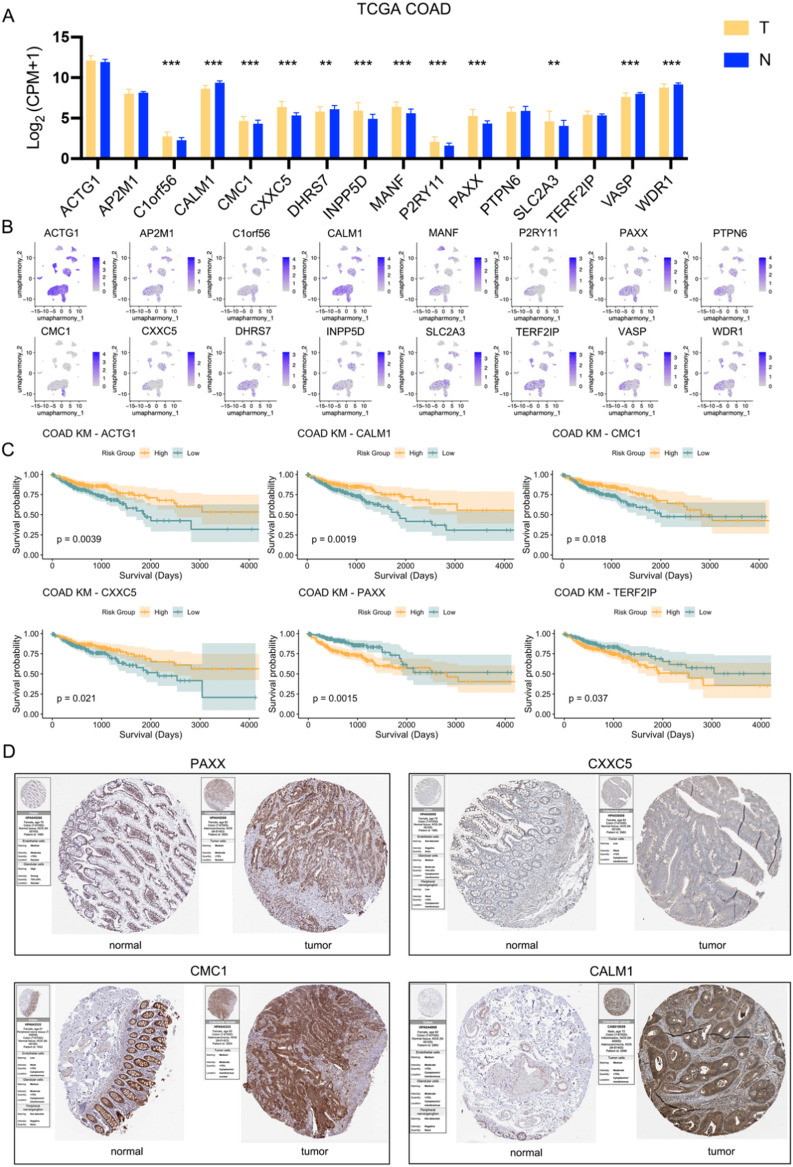
Validation of NK cell-associated prognostic genes. (**A**) Expression levels of prognostic genes in tumor versus normal tissues. **, *P* < 0.01; ***, *P* < 0.001; (**B**) Distribution of prognostic genes across cell subtypes; (**C**) KM survival analysis of prognostic genes; (**D**) Immunohistochemical analysis of key prognostic genes.

Compared to normal tissues, the expression of prognostic genes *C1orf56*, *CMC1*, *CXXC5*, *INPP5D*, *MANF*, *P2RY11*, *PAXX*, and *SLC2A3* was upregulated in tumor tissues, while *CALM1*, *DHRS7*, *VASP*, and *WDR1* were downregulated (Fig. 4A). Analysis of gene distribution across cell subtypes revealed enrichment not only in NK cells but also in other cell types (Fig. 4B). Genes *TERF2IP*, *PAXX*, *CXXC5*, *CMC1*, *CALM1*, and *ACTG1* demonstrated strong performance in predicting patient outcomes (Fig. 4C). Genes with both differential expression and prognostic value (PAXX, CXXC5, CMC1, CALM1) were validated via immunohistochemistry (Fig. 4D).

### Construction and validation of a pathology-based prognostic model

**Fig. 5:**
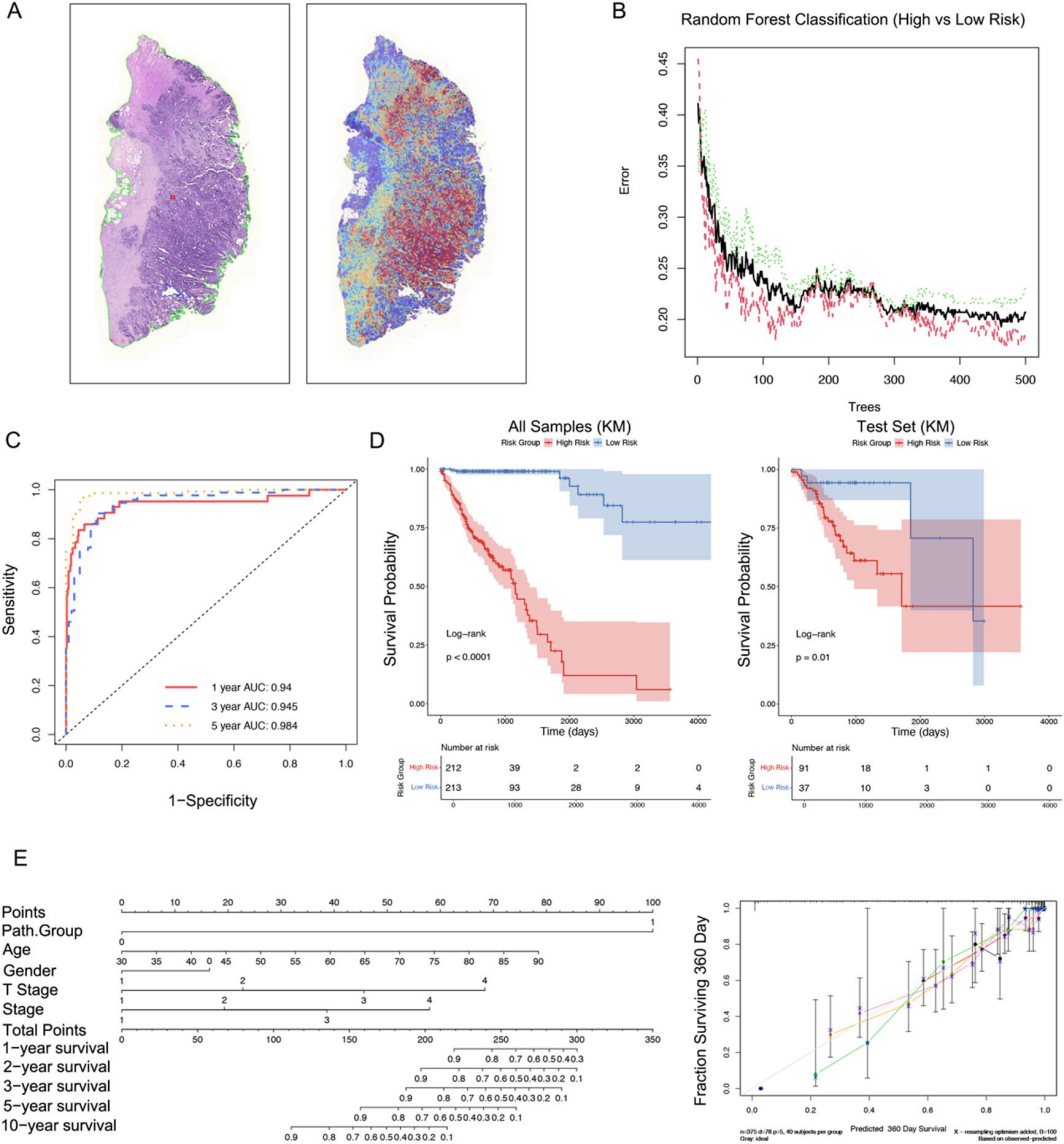
Deep neural network and pathology-based prognostic model construction. (**A**) Attention heatmap of COAD histopathological slides, with high-attention regions displayed in red and low-attention regions in blue; (**B**) Trend of error rate with increasing number of decision trees in the random forest classification model for high-risk and low-risk groups; (**C**,** D**) ROC and KM analyses for pathology-based high-risk and low-risk groups; (**E**) Construction of a nomogram based on pathological features.

We retrieved 458 histopathological images of COAD patients from TCGA. WSIs were processed using the CLAM tool. To realize the visualization and interpretation of each region’s relative importance in WSIs, attention scores for predicted categories were first converted into percentile-normalized values. Subsequent mapping of these values onto the original slides allowed for the generation of attention heatmaps (Fig. 5A). Using ResNet50, 1,024 pathological features were extracted from the slides. Univariate Cox regression identified 54 pathological features significantly associated with OS. A random survival forest (RSF) model was employed for survival risk analysis, with data split into training (70%) and testing (30%) sets for survival prediction modeling (Fig. 5B). ROC curve analysis validated the model’s predictive performance, with an AUC > 0.940 for the entire cohort, indicating excellent discrimination of survival status (Fig. 5C). Based on the median predicted risk score, samples were divided into low-risk and high-risk pathology groups, with the high-risk group exhibiting significantly worse prognosis (Fig. 5D). A nomogram that incorporates both pathological risk groups and clinical data was constructed, aiming to present predictive variables along with their corresponding weights. The nomogram model achieved a C-index of 0.871, with calibration curves showing consistent agreement between predicted and observed outcomes, confirming high predictive accuracy (Fig. 5E).

### Construction and efficacy validation of multimodal prognostic model

**Fig. 6:**
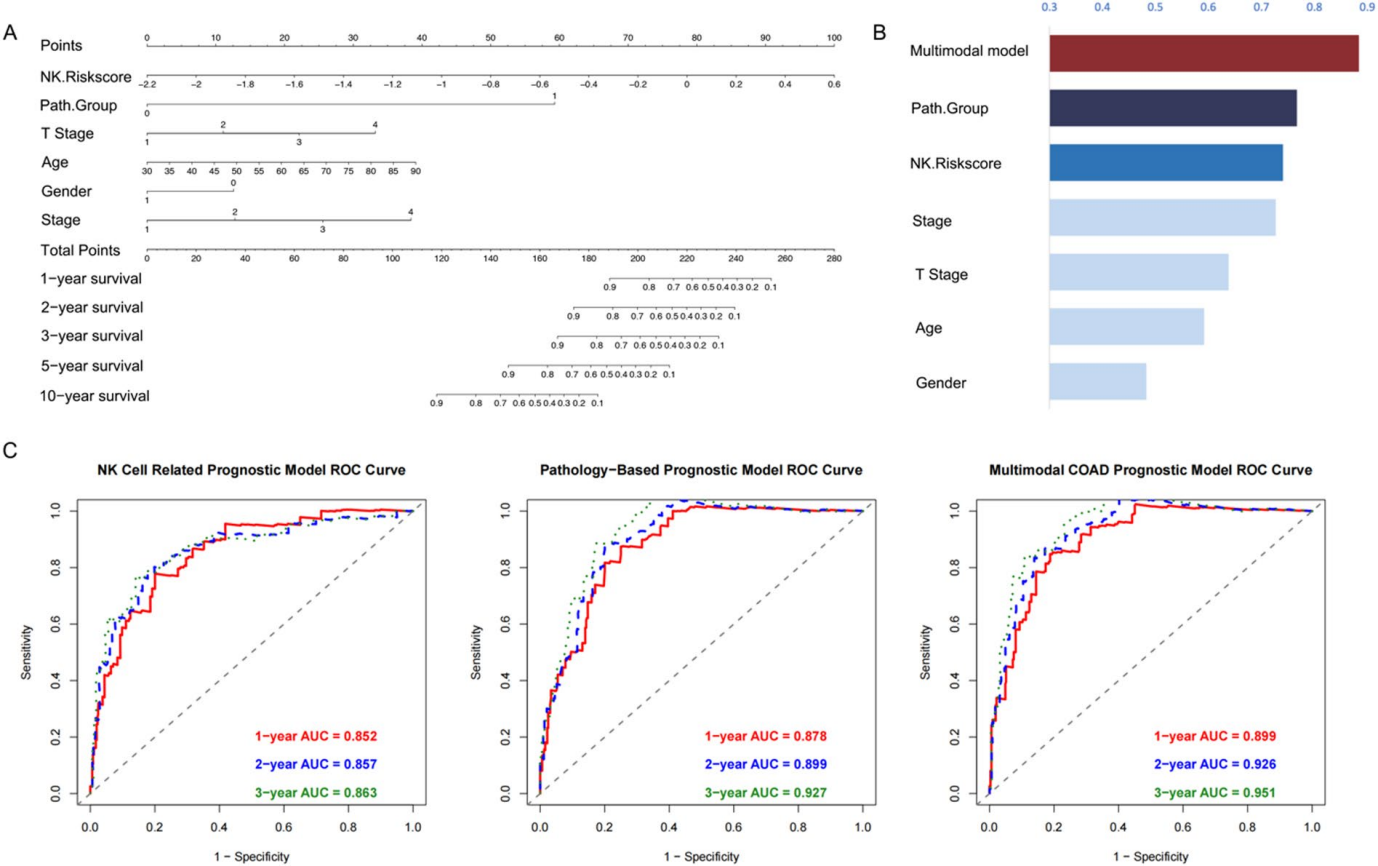
Construction of a multimodal prognostic model for COAD. (**A**) Development of a multimodal nomogram; (**B**) Evaluation of the multimodal prognostic model using the C-index; (**C**) The ROC shows that the multimodal model maintains high predictive accuracy at all follow-up time points.

Finally, a multimodal prognostic model for COAD was developed, integrating NK cell-associated risk scores, pathology-based risk groups, age, sex, T stage, and tumor stage (Fig. 6A). The model achieved a C-index of 0.889, demonstrating improved predictive performance compared to previous models (Fig. 6B). Statistical validation results showed that the C-index of the multimodal model was 0.889 (95%CI: 0.843–0.907, SE = 0.016), which was significantly higher than that of the NK- associated risk model (0.835, 95%CI: 0.772–0.869, SE = 0.023) and the pathology-related risk model (0.871, 95%CI: 0.829–0.894, SE = 0.016). To formally evaluate whether the performance differences between models were statistically significant, concordance indices were compared using the compareC method. The results demonstrated that the multimodal model significantly outperformed the NK-based model (ΔC-index = 0.054, *P* = 0.002), the pathology-based model (ΔC-index = 0.018, *P* = 0.025), and the clinical model (ΔC-index = 0.110, *P* < 0.001) (Table 1). Only 38 patients, about 10% of the cohort, were followed for more than 1825 days (5 years). This results in a sparse risk set at later times. In this case, rare extreme AUC values may occur. Therefore, to avoid potential bias due to sparse follow-up data at later times, we only present AUC values at 1, 2, and 3 years (Table S1). In terms of time-dependent AUC comparison, the multimodal model maintained the highest AUC value at all follow-up time points: 1-year (0.899), 2-year (0.926), 3-year (0.951) (Fig. 6C). These results suggest that the integration of molecular and histopathological features provides complementary prognostic information, contributing to the improved performance of the multimodal framework. This integration of scRNA-seq, transcriptomics, clinical data, and pathomics successfully established a multimodal prognostic model for COAD.

**Table 1:**
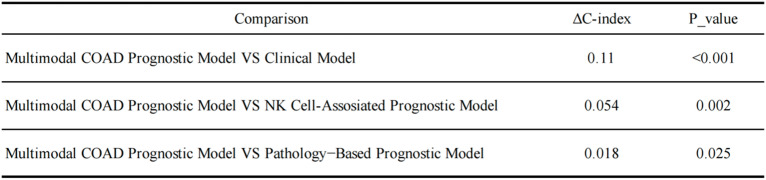
Comparative Analysis of Predictive Performance Among Multimodal and Single-Modal Prognostic Models.

## Discussion

COAD exhibits a rising incidence among digestive system cancers, remaining a highly lethal malignancy with a low 5-year survival rate despite advancements in di-agnostic and therapeutic technologies^42,43^. Early diagnosis and accurate prognostic prediction are critical research priorities. Single-cell omics and pathomics offer novel molecular insights for COAD early detection. Single-cell RNA sequencing elucidates the cellular heterogeneity and functional characteristics of the tumor microenvironment, facilitating identification of early pathological changes. Pathomics, leveraging deep learning algorithms, extracts key features from whole-slide images, enhancing diagnostic objectivity and efficiency for COAD screening^44,45^. This study integrates single-cell RNA sequencing, transcriptomics, pathomics, and clinical data to characterize COAD tumor microenvironment heterogeneity, develop immune cell-related prognostic models, and establish a multimodal prognostic framework.

Single-cell RNA sequencing delineated the composition and functional roles of diverse cell subpopulations within the COAD tumor microenvironment, particularly their contributions to anti-tumor immunity^46^. Significant inter-patient variability in immune cell proportions suggests individualized microenvironment characteristics that may influence treatment response and prognosis^47^. A prognostic model constructed using the TCGA-COAD cohort, incorporating LASSO regression and Cox analysis, identified key immune-related genes with predictive value for risk stratification. GSVA revealed distinct metabolic, immune, and tumor-related pathway differences between high-risk and low-risk groups, underscoring the model’s biological relevance. Functional enrichment analysis revealed that the identified genes were associated with several immune-related pathways, including NF-kappa B signaling pathway, MAPK, and Rap1 signaling pathways. These pathways are known to regulate immune activation, inflammatory signaling, and tumor–immune interactions. Dysregulation of these pathways may contribute to immune dysfunction or immune escape in high-risk COAD patients. Therefore, the NK-associated gene signature may reflect a tumor microenvironment characterized by altered immune signaling and impaired anti-tumor immune responses. KM curves and ROC analyses demonstrated high predictive accuracy, with nomograms confirming clinical applicability. The high-risk group exhibited elevated Tumor Immune Dysfunction and Exclusion scores, indicating greater immune escape potential and potentially reduced responsiveness to immune checkpoint inhibitors^48^. A deep learning model extracted diagnostically significant features from whole-slide images, generating attention heatmaps to visualize critical pathological regions, improving diagnostic efficiency^49^. The improved performance of the multimodal model suggests that integrating molecular and histopathological information provides complementary prognostic value rather than being dominated by a single modality.

The methodological innovations of this study are multifaceted. First, scRNA-seq enabled single-cell resolution analysis of the TME, revealing the heterogeneity and functional characteristics of immune cell subpopulations. Second, LASSO regression and DL algorithms were employed to analyze transcriptomic and pathomic data, con-structing multiple high-risk precision prognostic models. Finally, the multimodal framework, integrating single-cell, transcriptomic, and pathomic features, overcame the limitations of single data types, providing novel tools for precision medicine. These approaches collectively enhance the understanding of COAD molecular mechanisms and establish a foundation for clinical translation^50^. The TNM staging system remains the standard clinical tool for colorectal cancer prognosis but primarily reflects anatomical tumor burden and may not fully capture tumor biological heterogeneity. Immune score provides prognostic information based on immune cell infiltration within the tumor microenvironment, while the CMS classification stratifies tumors into molecular subtypes with distinct biological and clinical characteristics. In comparison, our model integrates NK cell–associated molecular features with histopathological and clinical information, aiming to capture complementary aspects of tumor biology and the immune microenvironment. Therefore, rather than replacing existing systems, the proposed model may serve as a complementary prognostic tool that integrates multi-dimensional biological information beyond traditional staging approaches. With larger-scale validation and methodological refinements, these findings hold promise for advancing the understanding of COAD and laying the foundation for innovative diagnostic and therapeutic approaches.

Despite these advances, the study has limitations. First, the sample size for scRNA-seq is limited , which may affect the representativeness and generalizability of the results^51,52^. It should be noted that NK cell-associated genes may also be expressed in other cell populations in the tumor microenvironment, implying that the prognostic risk score reflects the overall immune-tumor microenvironment status rather than NK cell activity alone. In this study, the single-cell RNA sequencing analysis was primarily used as a hypothesis-generating and feature-selection step to identify candidate NK cell–associated genes within the tumor microenvironment, with the main objective of providing candidate molecular features for downstream prognostic modeling. Although the GSE161277 dataset is a scarce single-cell dataset in the COAD field that includes both normal and tumor tissues with complete clinical information and has been validated and applied in multiple high-quality studies, and 12,042 high-quality cells were obtained after quality control with cell coverage and gene detection depth meeting the basic requirements of single-cell transcriptome analysis, the small sample size may still fail to capture some rare NK cell subpopulations, and the generalizability of the gene signatures needs to be validated in larger cohorts. We will address this by expanding the analysis with multi-center datasets in the future. Second, external validation of the prognostic model relied on a single dataset (GSE17538), necessitating broader validation to confirm its robustness^53^. In the external validation cohort, the model achieved time-dependent AUC values of 0.57, 0.57, and 0.56 at 1, 3, and 5 years, respectively. The relatively lower performance compared with the TCGA cohort may be related to differences in gene expression platforms, patient population heterogeneity, and the smaller sample size of the external datasets. Despite this slight decrease in performance, the P value of the externally validated KM survival analysis was still less than 0.05, confirming that the model had some generalization ability. We will further validate it in the future by including more datasets from multiple centers and different detection platforms. Third, the CLAM attention heatmap was used primarily as an interpretable tool to assist pathologists in identifying potentially informative tumor regions in this study. Because these attention maps are generated by the model and are not designed as precise localization outputs, quantitative validation of specific regions is challenging. And the model’s dependence on high-quality pathological images may limit its performance, as variations in data format or quality could introduce confounding factors^54,55^.

In terms of the clinical transformation application prospects of the model, this study proposes the following practical application paths: For newly diagnosed COAD patients, CLAM model analysis can first be performed using routine pathological sections—widely used in clinical practice—to quickly obtain pathomics risk scores; simultaneously, gene risk scores can be obtained by detecting 16 NK cell-associated core genes via RT-qPCR in peripheral blood or tumor tissues, a method that enables high-throughput and low-cost detection; finally, integrate clinical data such as age and T stage, and quickly calculate patients’ 1-year, 3-year, and 5-year survival probabilities through nomograms to achieve risk stratification. In terms of cost-benefit analysis, pathomics analysis is based on existing pathological sections, requiring no additional sampling and only increasing the cost of computing resources. Detecting 16 NK cell-associated core genes to obtain gene risk scores features low detection costs and high feasibility. The model can help identify high-risk patients, guiding them to receive more intensive follow-up or combined immunotherapy to reduce the risk of late recurrence; for low-risk patients, it can reduce unnecessary treatment interventions, alleviating the medical burden. Overall, the model boasts a favorable cost-benefit ratio.

Future research could focus on integrating spatial transcriptomics to characterize the spatiotemporal dynamics of the TME and validate the spatial distribution of cell sub-populations, expanding the scale and diversity of external validation datasets to enhance model generalizability, conducting in vivo functional experiments to explore the bio-logical roles of key genes, and incorporating additional omics data to further improve prognostic accuracy^56–59^. These efforts will advance the clinical application of precise COAD diagnosis and personalized treatment.

## Supplementary Information

Below is the link to the electronic supplementary material.

Supplementary file1 (DOC 1270 KB)

## Data Availability

The single-cell RNA sequencing dataset analyzed in this study is available in the Gene Expression Omnibus (GEO) repository under accession number GSE161277. The independent gene expression dataset used for external validation is available in the GEO repository under accession number GSE17538. Bulk gene expression data, corresponding clinical information, and whole-slide images (WSIs) for colon adenocarcinoma were obtained from The Cancer Genome Atlas (TCGA) database. Immunohistochemical staining data used to verify protein expression patterns were obtained from the Human Protein Atlas (HPA) database. All datasets used in this study are publicly available and accessible without restriction for research purposes.

## Code Availability

The analysis code used in this study will be publicly available via the GitHub repository after publication to facilitate reproducibility of the analysis. DOI: 10.5281/ Zenodo.19050569. The code base is publicly accessible and will be maintained in perpetuity to ensure long-term availability and traceability of research methods.

Statistical Analysis.

All analyses were performed using R (version 4.3.1) or Python (version 3.7.12), with statistical significance set at *p* < 0.05 for all tests.
