# Supplementary material for: A multimodal approach integrating NK cell-associated gene signatures and pathomics to predict colon adenocarcinoma prognosis
Source: Sci Rep. 2026 Apr 22;16:18641. doi: 10.1038/s41598-026-49584-y (PMC13269797; doi:10.1038/s41598-026-49584-y)
Supplement: Supplementary file 1 — Supplementary file1 (DOC 1270 KB) [file 41598_2026_49584_MOESM1_ESM.docx]

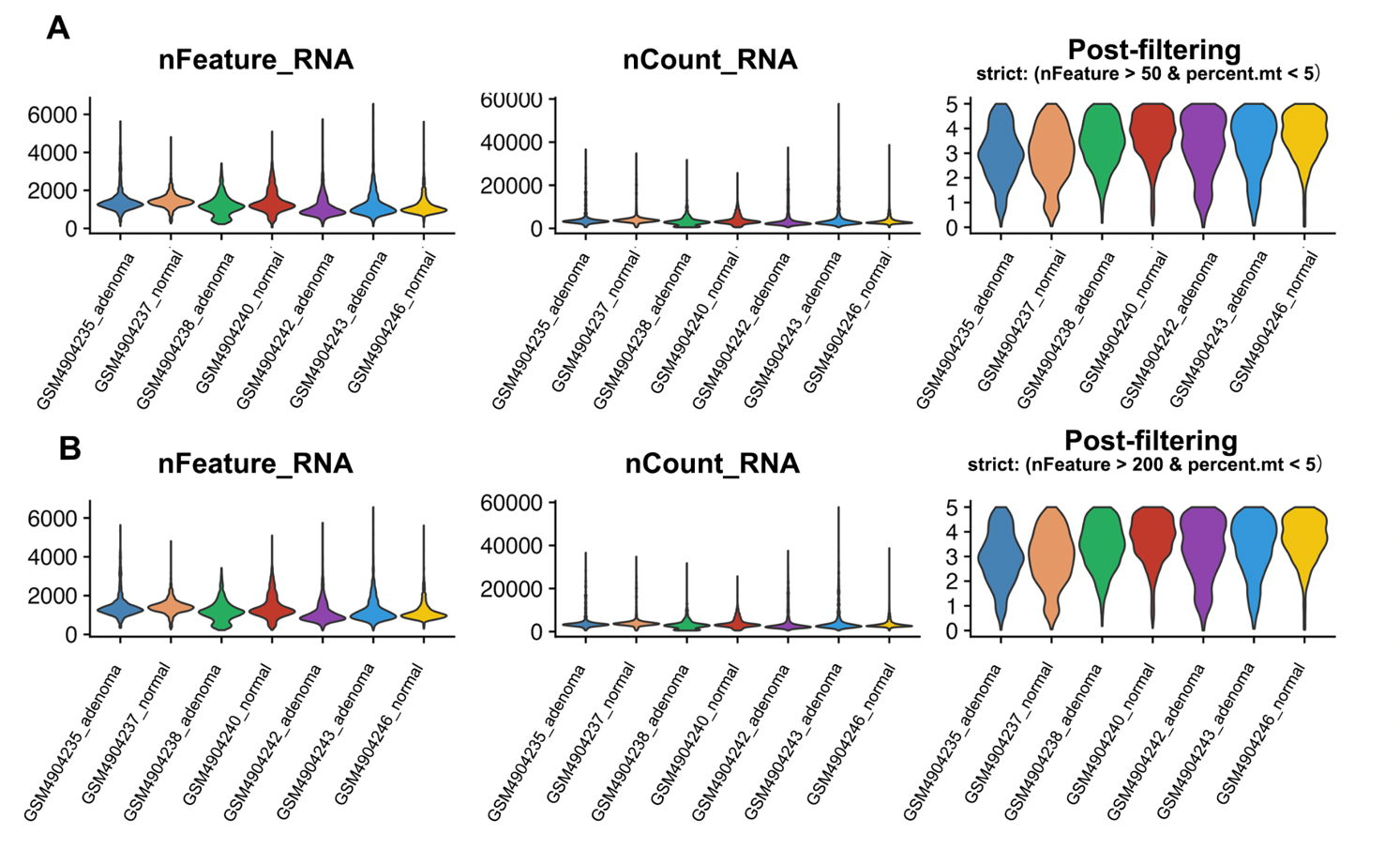


Figure S1. Quality control and sensitivity analysis of single-cell data filtering thresholds. **(A)** Quality control metrics using permissive filtering thresholds (nFeature_RNA > 50, percent.mt < 5%); **(B)** Quality control metrics using strict filtering thresholds (nFeature_RNA > 200, percent.mt < 5%)


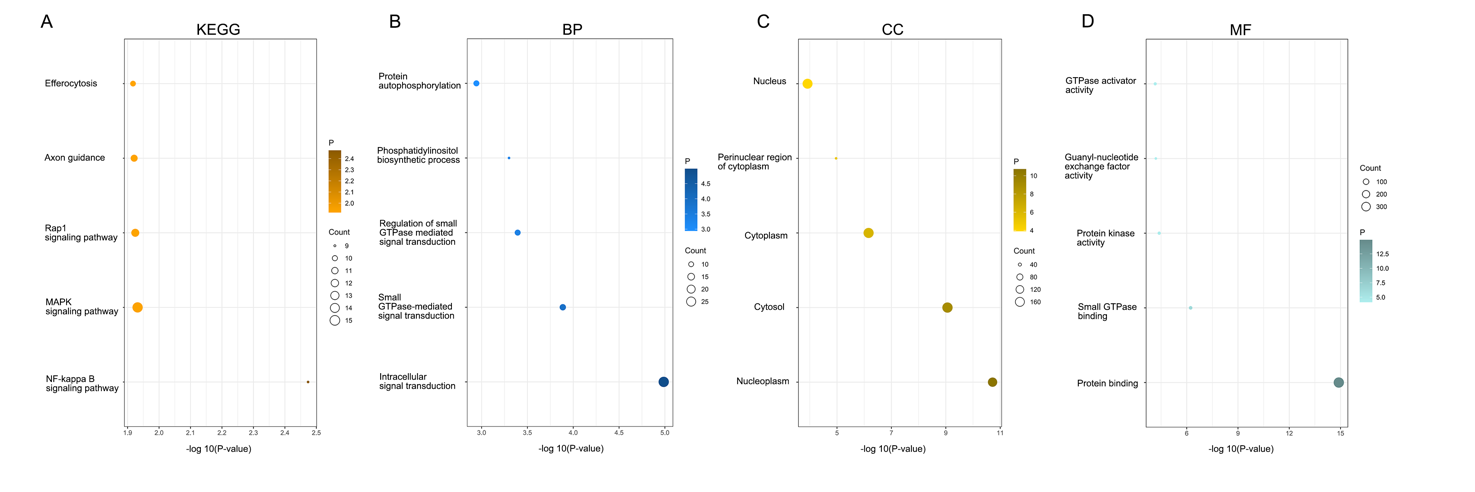


Figure S2. Functional Enrichment Analyses of NK Cell-associated Prognostic Genes. **(A)** KEGG pathway enrichment analysis; **(B)** Biological Process (BP) enrichment analysis; **(C)** Cellular Component (CC) enrichment analysis; **(D)** Molecular Function (MF) enrichment analysis


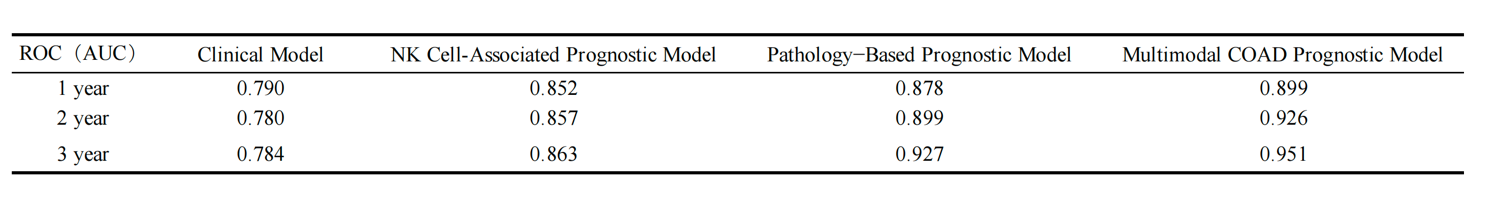


Table S1. Time-dependent AUC values of different models at 1, 2, and 3 years for COAD prognosis.
